# Supplementary material for: Conformational landscape of HIV-1 Env from closed to fully open
Source: Nat Commun. 2026 Feb 24;17:4273. doi: 10.1038/s41467-026-69921-z (PMC13168245; doi:10.1038/s41467-026-69921-z)
Supplement: Supplementary file 1 — Supplementary Information [file 41467_2026_69921_MOESM1_ESM.pdf]

Supplementary Information

Conformational Landscape of HIV-1 Env from Closed to Fully Open

Jiayan Cui, Zi Jie Lin, Sukanya Ghosh, Jianqiu Du, Roopak Sadeesh, David B. Weiner & Jesper Pallesen

Supplementary Figures 1-7	Pages 2-9
Supplementary Tables 1-2	Pages 10-11
Supplementary Movies 1-3	

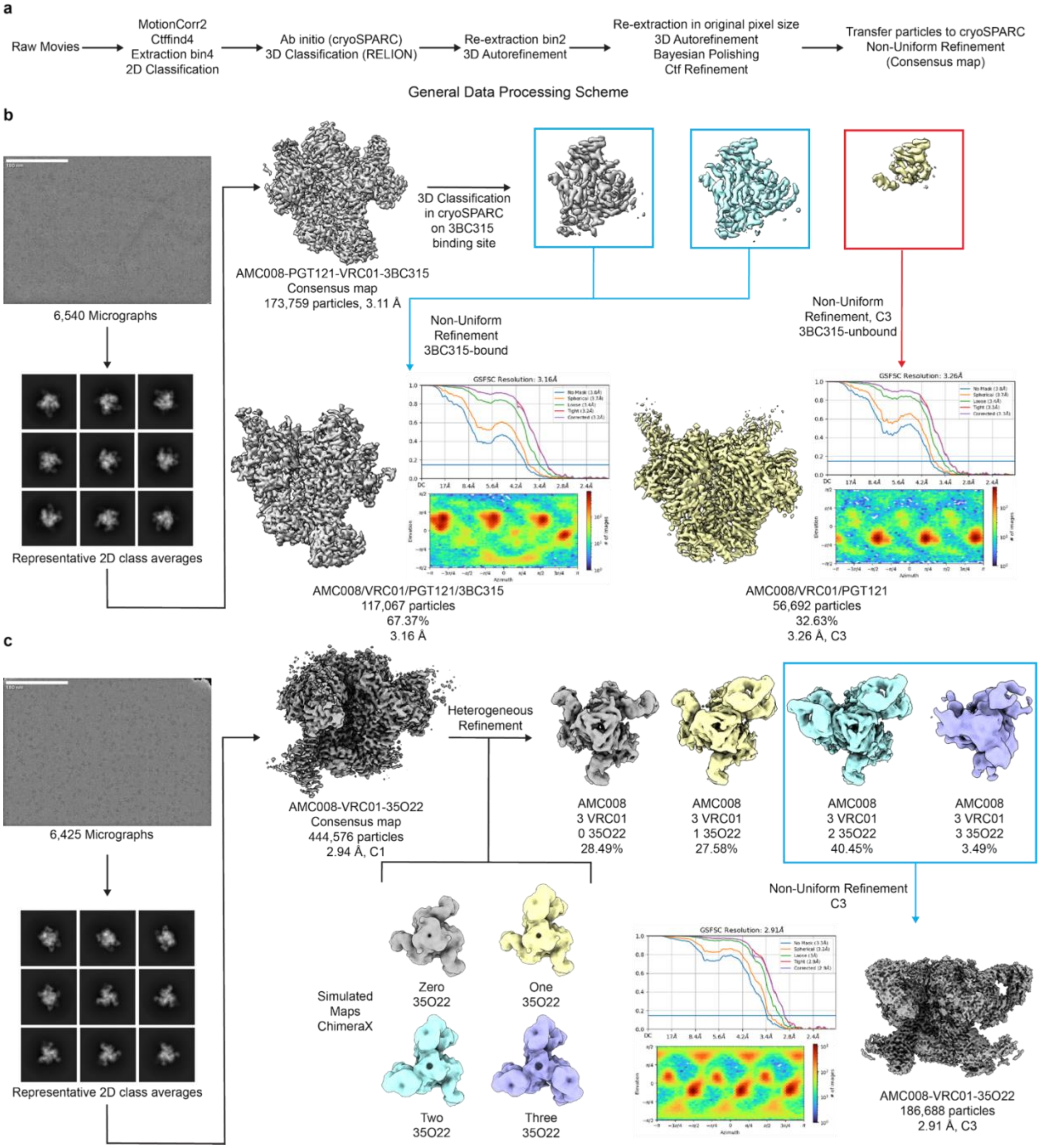

Supplementary Figure 1 | Cryo-EM data processing scheme I. a General cryo-EM data processing scheme to generate consensus maps in this study. **b** Additional Cryo-EM data processing for AMC008 in complex with PGT121, VRC01, and 3BC315. Particles used for the reconstruction of the consensus map were 3D classified focused on the 3BC315 binding site. Classes that have 3BC315 binding were combined and used for Non-Uniform Refinement. **c** Additional Cryo-EM data processing for AMC008 in complex with VRC01 and 35O22. Simulated maps generated using ChimeraX were used as templates for Heterogeneous Refinement using particles from the consensus map of AMC008-VRC01-35O22. Classes with two and three 35O22 occupancy were combined and used for Non-Uniform Refinement with C3 symmetry.

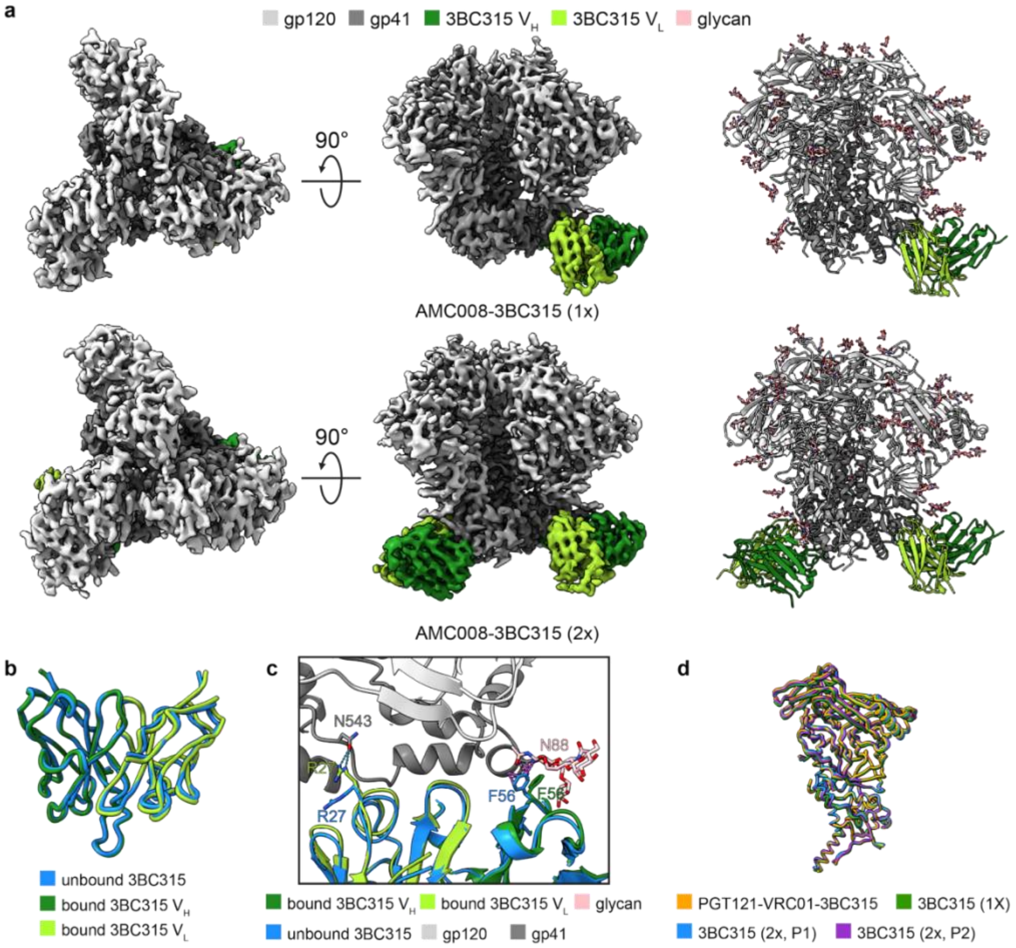

Supplementary Figure 2 | Additional data of 3BC315-bound AMC008 SOSIP. a 3.6 Å and 3.5 Å cryo-EM density maps (left and middle, top and side views) and atomic models (right) of AMC008 in complex with one 3BC315 Fab (top) and two 3BC315 Fabs (bottom). **b** Comparison of AMC008 bound 3BC315 to unliganded 3BC315 crystal structure (5CCK). **c** Conformational changes of 3BC315 after binding to AMC008. **d** Comparison of 3BC315 liganded AMC008 protomers in AMC008-PGT121-VRC01-3BC315, AMC008-3BC315 (1x), and AMC008-3BC315 (2x). P1, 3BC315-bound Protomer 1; P2, 3BC315-bound Protomer 2.

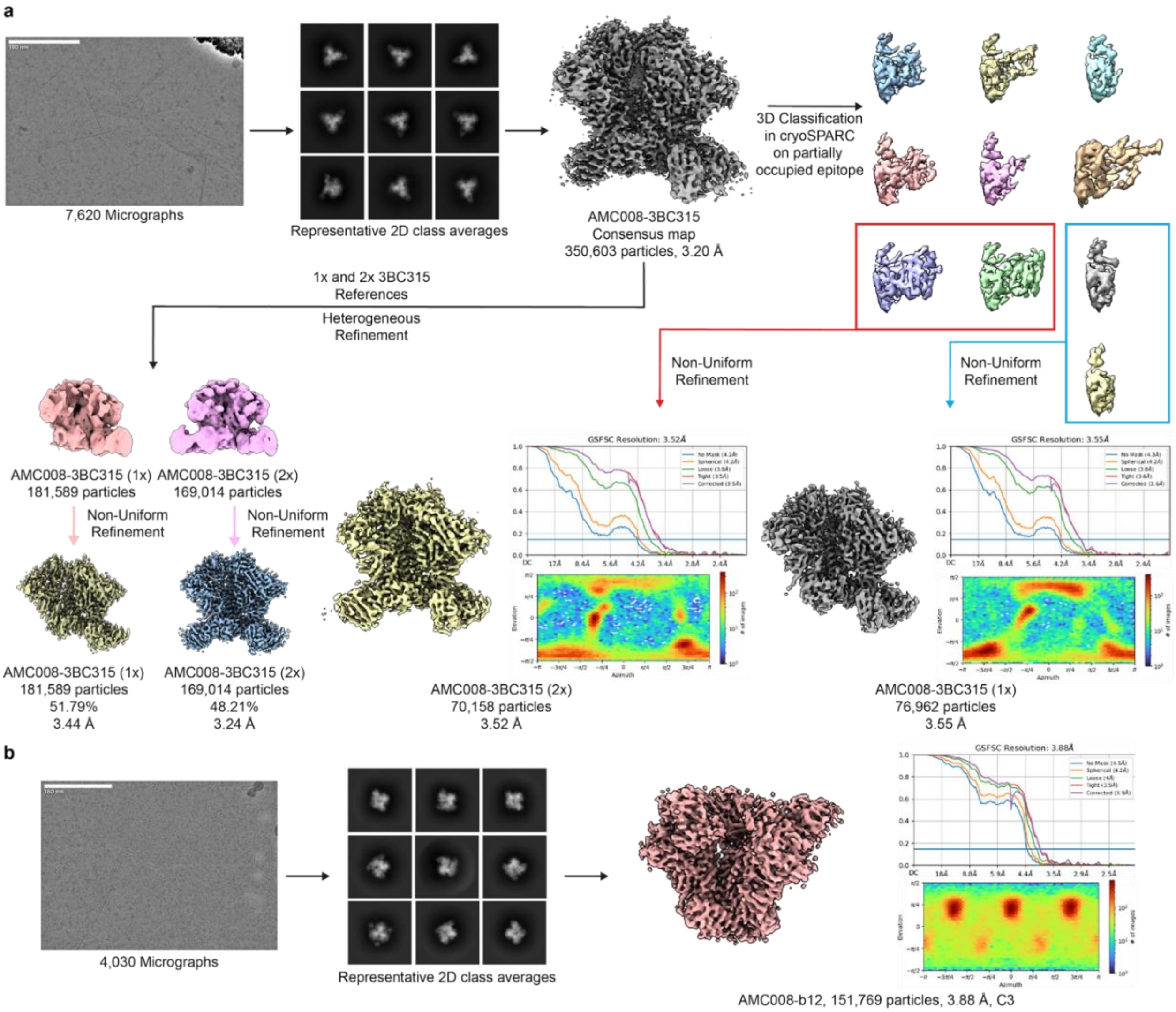

Supplementary Figure 3 | Cryo-EM data processing scheme II. a Additional Cryo-EM data processing for AMC008 in complex with 3BC315. Particles used for the reconstruction of the consensus map were 3D classified, followed by Non-Uniform Refinement to generate AMC008 Envs occupied with one 3BC315 and two 3BC315. Maps of AMC008 bound by one and two 3BC315 were used as templates for Heterogeneous Refinement. **b** Density map of AMC008 in complex with b12, along with their GS-FSC curve, resolution, and particle distribution plot.

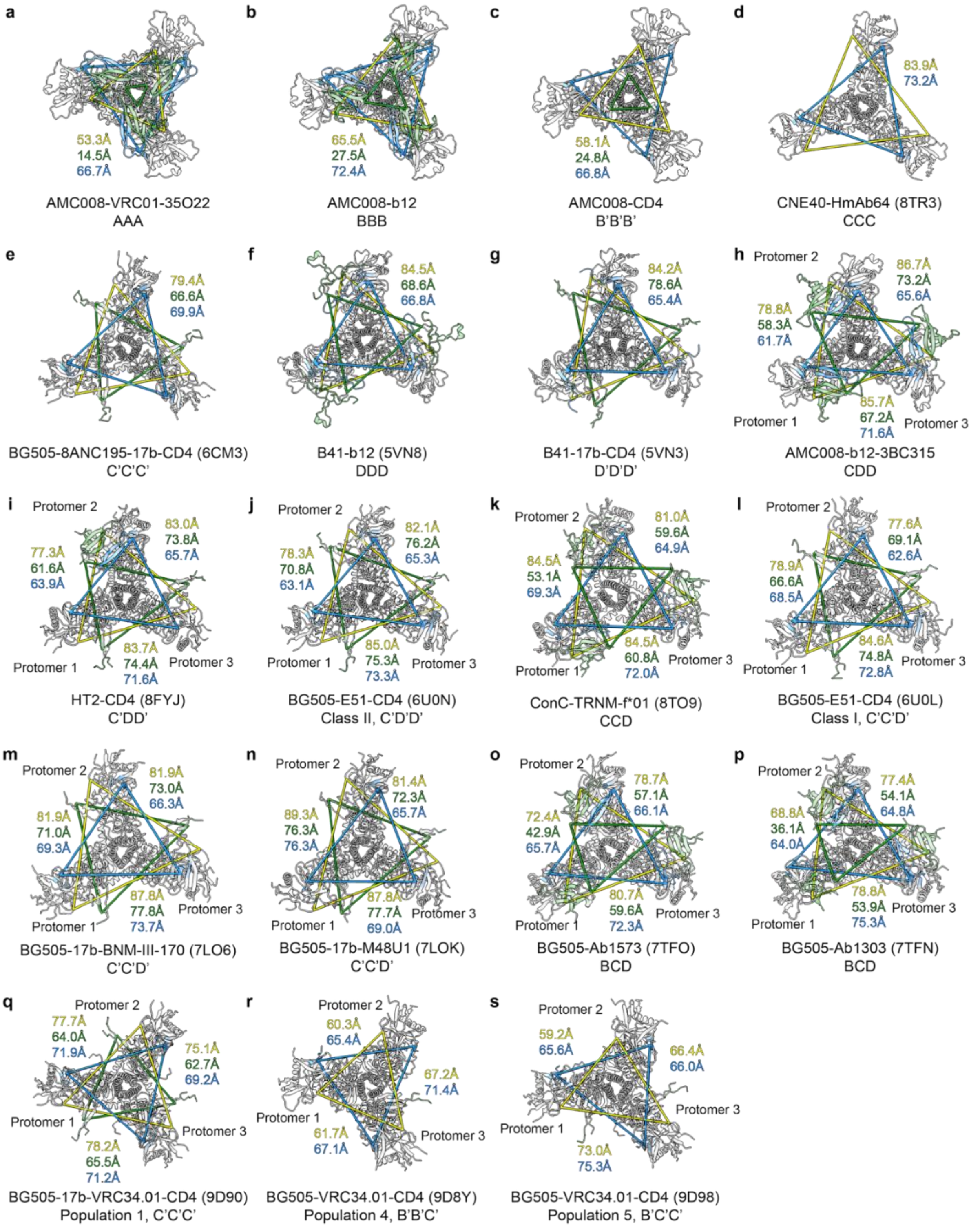

(legend on next page)

Supplementary Figure 4 | Measurement of interprotomer distances. **a**-**s** Measurement of interprotomer distances of V1V2 apex (Pro124, green), V3 (Cys296, blue) and, CD4bs (Asp368, yellow) in closed (AMC008-VRC01-35O22, **a**), b12-bound moderately open (AMC008-b12, **b**), CD4-bound moderately open (AMC008-b12, **c**), HmAb64-bound partially open (CNE40-HmAb64, **d**), CD4-bound partially open (BG505-8ANC195-17b-CD4, **e**), b12-bound fully open (B41-b12 ,**f**), CD4-bound fully open (B41-17b-CD4, **g**), b12 and 3BC315-bound asymmetric open (AMC008-b12-3BC315, **h**), two CD4 bound asymmetric open (HT2-CD4, **i**), three CD4-bound asymmetric open (BG505-E51-CD4, Class I and II, **j** and **l**), TRNM-f*01-bound asymmetric open (ConC-TRNM-f*01, **k**), BNM-III-170-bound asymmetric open (BG505-17b-BNM-III-170, **m**), M48U1-bound asymmetric open (BG505-17b-M48U1, **n**), Ab1303-bound asymmetric open (BG505-Ab1573, **o**), Ab1573-bound asymmetric open (BG505-Ab1303, **p**), CD4-bound asymmetric open (BG505-17b-VRC34.01-CD4, population 1, **q**; BG505-VRC34.01-CD4, population 4 and 5, **r** and **s**) Env models. V1 and V2 loops are colored in green; V3 loop is colored in blue; gp120 is colored in light gray; gp41s is colored in gray. Fabs and CD4s are hidden for better visualization. Closed states are denoted with A, moderately open states are denoted with B, partially open states are denoted with C, fully open states are denoted with D, CD4-bound states are denoted with ’. Pro124 residues are not ordered in **c** and **r**-**s**.

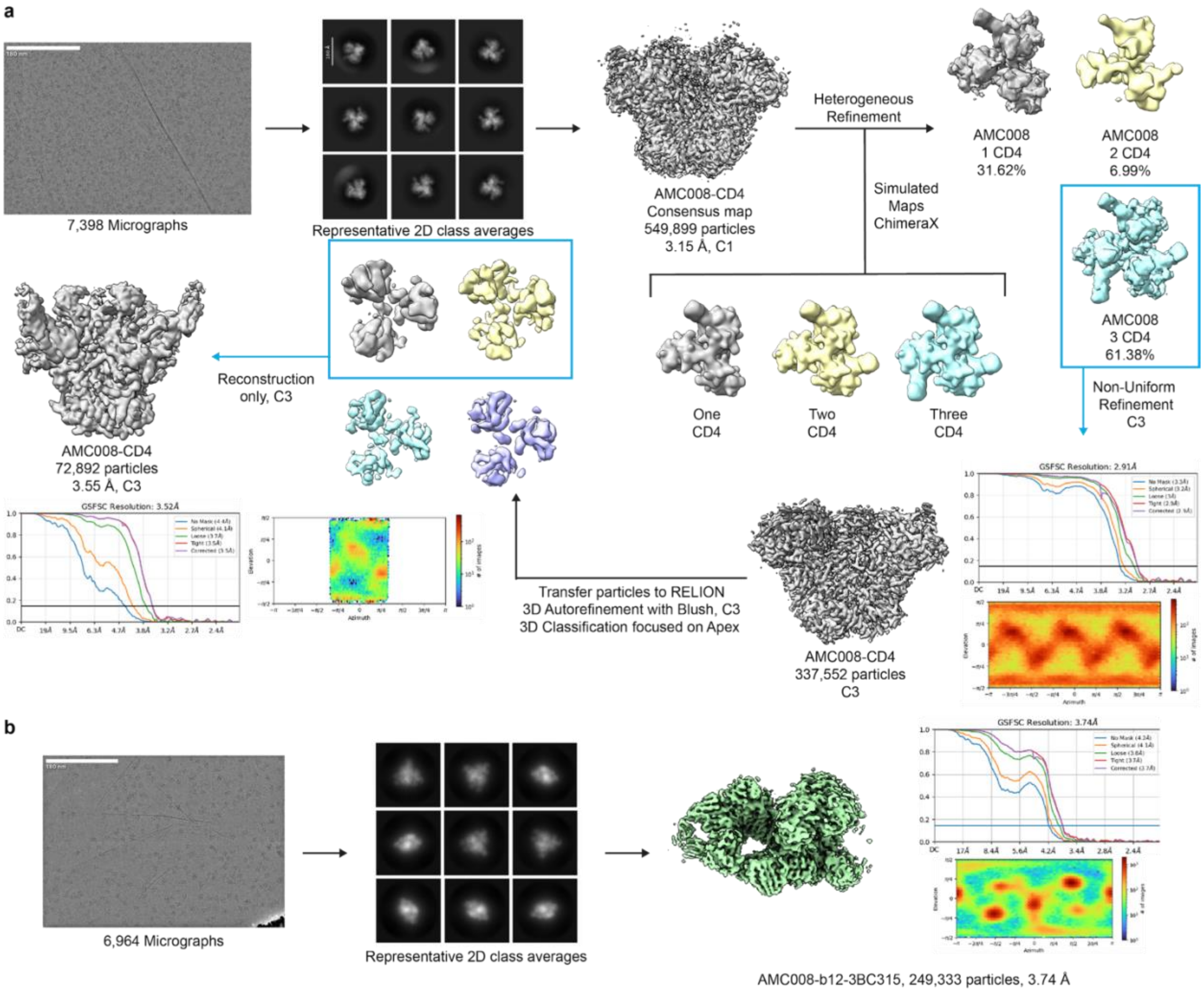

Supplementary Figure 5 | Cryo-EM data processing scheme III. a Additional Cryo-EM data processing for AMC008 in complex with CD4. Simulated maps of AMC008 bound by one, two, and three CD4 were generated using ChimeraX and used as templates for Heterogeneous Refinement using particles from the consensus map of AMC008-CD4. The class with three CD4 occupancy was used for Non-Uniform Refinement with C3 symmetry to reconstruct the map of AMC008 in complex with three CD4. The map of AMC008 in complex with three CD4 was 3D classified focusing on the Env apex with four classes. The two classes with density definition on Env apex were combined and imposed C3 symmetry to reconstruct a map with better density on Env apex. **b** Density map of AMC008 in complex with b12 and 3BC315, along with their GS-FSC curve, resolution, and particle distribution plot.

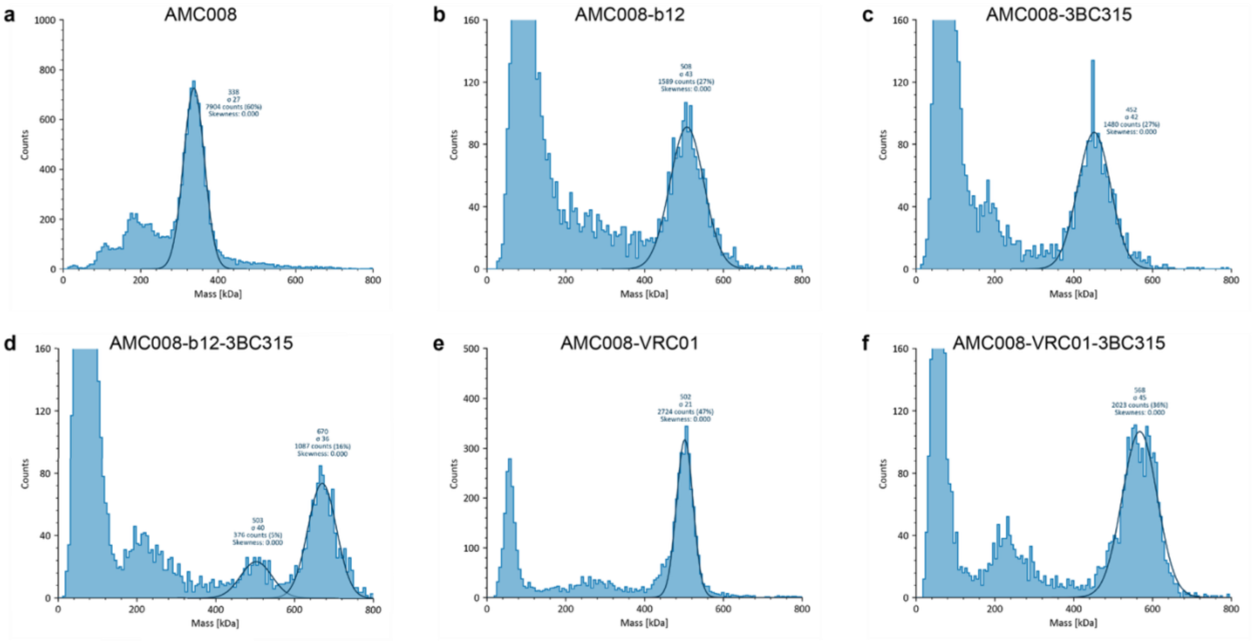

Supplementary Figure 6 | Mass photometry experiments of AMC008 in complex with Fabs. a-**f** Mass histograms of AMC008 (**a**) and AMC008 in complex with excess b12 (**b**), 3BC315 (**c**), b12 and 3BC315 (**d**), VRC01 (**e**), VRC01 and 3BC315 (**f**).

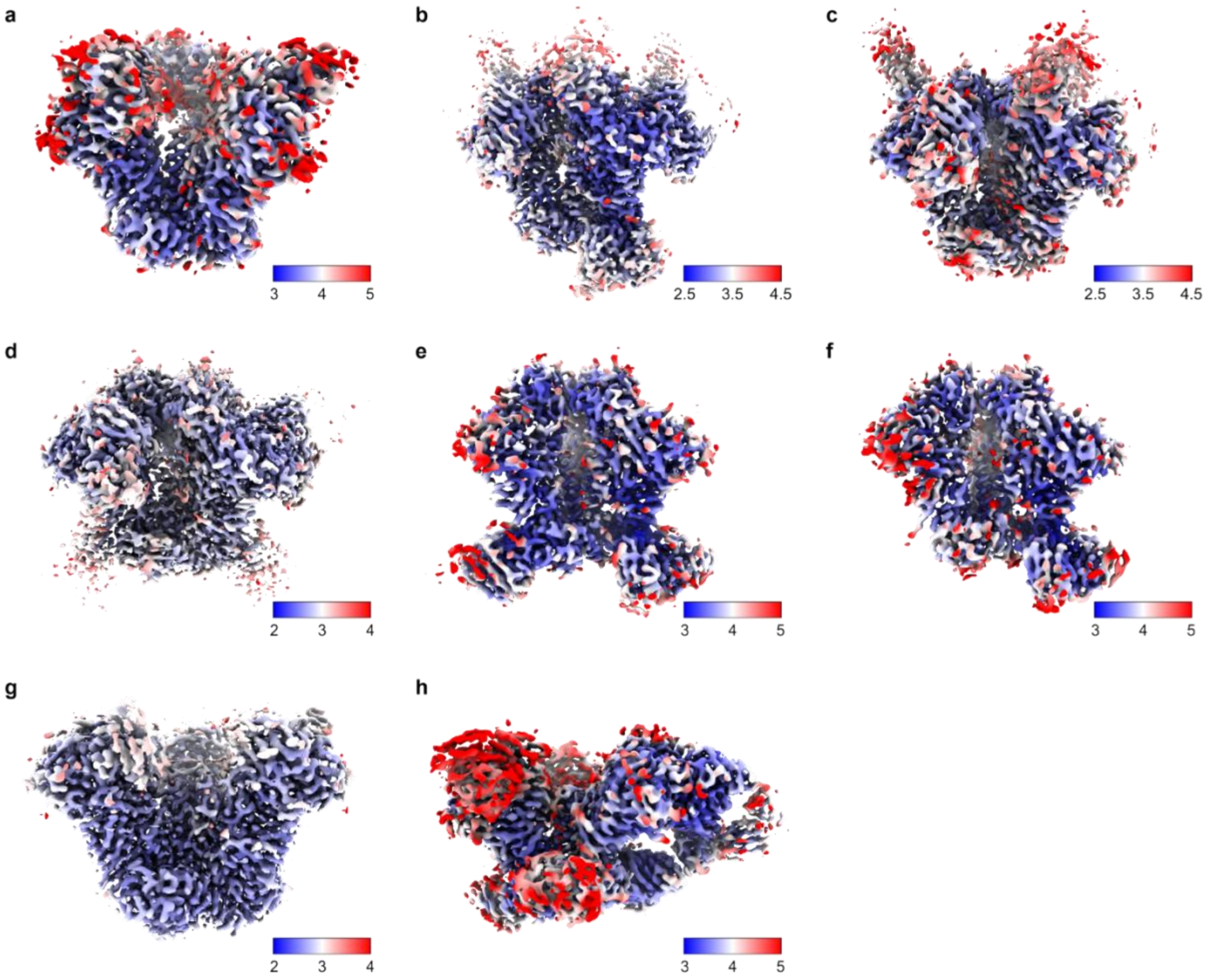

Supplementary Figure 7 | Local resolution estimation for the cryo-EM density maps of AMC008 in complex with Fabs and CD4. a-h Side views of the cryo-EM density maps of AMC008 in complex with three b12 Fabs (**a**); three PGT121, three VRC01 and one 3BC315 Fabs (**b**); three PGT121 and three VRC01 Fabs (**c**); three VRC01 and 35O22 Fabs (**d**); two 3BC315 Fabs (**e**); one 3BC315 Fab (**f**); three CD4s (**g**); three b12 and 3BC315 Fabs (**h**). Maps are colored by local resolutions.

PDB ID	Env	Ligands	Symmetry	State	Note
9NBY	AMC008 SOSIP.v4.2	VRC01, PGT121, 3BC315	C1	$A_{BR}AA$	This study
9NC8	AMC008 SOSIP.v4.2	3BC315	C1	$A_{BR}AA$	This study
9NC6	AMC008 SOSIP.v4.2	3BC315	C1	$A_{BR}A_{BR}A$	This study
6NQD	T/F100 SOSIP	8ANC195	C3	$A_{BR}A_{BR}A_{BR}$	
6VRW	CAP256.wk34.c80 SOSIP.RnS2	/	C3	$A_{BR}A_{BR}A_{BR}$	
6VTT	CAP256.wk34.c80 SOSIP.RnS2	CAP256-VRC26.25	C1	$A_{BR}A_{BR}A_{BR}$	
8FYI	BG505 SOSIP-HT1	CD4	C1	A'AA	
5U1F	BG505 DS-SOSIP	CD4, PGT145	C1	A'AA	
9NC0	AMC008 SOSIP.v4.2	b12	C3	BBB	This study
9OAJ	AMC008 SOSIP.v4.2	CD4	C3	B'B'B'	This study
9D8Y	BG505 SOSIP	CD4, VRC34.01	C1	B'B'C'	VRC34.01 binding remodels FP conformation
9D98	BG505 SOSIP	CD4, VRC34.01	C1	B'C'C'	VRC34.01 binding remodels FP conformation
7TFN	BG505 SOSIP	Ab1303	C1	BCD	
7TFO	BG505 SOSIP	Ab1573	C1	BCD	
8TR3	CNE40 SOSIP	HmAb64 scFv	C3	CCC	
6CM3	BG505 SOSIP	CD4, 17b, 8ANC195	C3	C'C'C'	
6EDU	B41 SOSIP	CD4, 21c, 8ANC195	C3	C'C'C'	
9D90	BG505 SOSIP	CD4, VRC34.01, 17b	C1	C'C'C'	VRC34.01 binding remodels FP conformation
8TO9	ConC SOSIP	TRNM-f*01	C3	CCD	C1 symmetry but map is reconstructed in C3
6U0L	BG505 SOSIP	CD4, E51	C1	C'C'D'	
7LO6	BG505 SOSIP	BNM-III-170, 17b	C1	C'C'D'	BNM-III-170 is a CD4 mimic
7LOK	BG505 SOSIP	M48U1, 17b	C1	C'C'D'	M48U1 is a CD4 mimic
9NC3	AMC008 SOSIP.v4.2	b12, 3BC315	C1	CDD	This study
8FYJ	BG505 SOSIP-HT2	CD4	C1	C'DD'	
6U0N	BG505 SOSIP	CD4, E51	C1	C'D'D'	
7LU9	CH505 SOSIP	DH851.3	C1	BDD	Low confidence due to the limited resolution
5VN8	B41 SOSIP	b12	C3	DDD	
5VN3	B41 SOSIP	CD4, 17b	C3	D'D'D'	
6OPN	B41 SOSIP	CD4, 17b, GO35	C3	D'D'D'	
6OPO	B41 SOSIP	CD4, 17b, DDM	C3	D'D'D'	
6X5B	B41 SOSIP	CD4, 17b, GO52	C3	D'D'D'	
7TXD	BG505 SOSIP	CD4, bnD.9	C3	D'D'D'	
8D5C	B41 SOSIP	CD4, CG10	C3	D'D'D'	
8Z7N	CH119 SOSIP	CD4	C3	D'D'D'	

Supplementary Table 1 | Summary of non-AAA Env structures classified by protomeric states in the PDB database. A, B, C, and D denote closed, moderately open, partially open, and fully open states. CD4-bound states are denoted with ’, base-relaxed states are denoted with BR. Fusion peptide conformations in BG505-CD4-VRC34.01 (states B’B’C’ and B’C’C’) and BG505-CD4-VRC34.01-17b (state C’C’C’) are remodeled by the binding of fusion peptide targeting antibody VRC34.01. Thus, the gp41 conformation of these complexes does not align with our model, but the gp120 openness, V1V2 displacement, formation of 4-stranded bridging sheet and α0 helix agree with states B’B’C’, B’C’C’, and C’C’C’.

	AMC008 SOSIP.v4.2 -b12 (EMDB-49239) (PDB 9NC0)	AMC008 SOSIP.v4.2 -PGT121 -VRC01 -3BC315 (EMDB-49238) (PDB 9NBY)	AMC008 SOSIP.v4.2 -PGT121 -VRC01 (EMDB-73342) (PDB 9YQO)	AMC008 SOSIP.v4.2 -VRC01 -35O22 (EMDB-49236) (PDB 9NBT)	AMC008 SOSIP.v4.2 -3BC315 (2x) (EMDB-49241) (PDB 9NC6)	AMC008 SOSIP.v4.2 -3BC315 (1x) (EMDB-49242) (PDB 9NC8)	AMC008 SOSIP.v4.2 -CD4 (EMDB-70287) (PDB 9OAJ)	AMC008 SOSIP.v4.2 -b12 -3BC315 (EMDB-49240) (PDB 9NC3)
**Data collection and processing**								
Magnification	81,000							
Voltage (kV)	300							
Electron exposure (e–/Å^2)	58							
Defocus range (μm)	0.5-2.5							
Pixel size (Å)	1.100	1.054						
Symmetry imposed	C3	C1	C3	C3	C1	C1	C3	C1
Final particle images (no.)	151,769	111,067	56,692	188,688	70,158	76,962	337,552	249,333
Map resolution (Å)	3.88	3.16	3.26	2.91	3.52	3.55	2.91	3.74
FSC threshold	0.143							
**Refinement**								
Initial model used (PDB code)	1HZH, 7SQ1	4LST, 4JY4, 5CCK, 7SQ1	4LST, 4JY4, 7SQ1	4LST, 4TOY, 7SQ1	5CCK, 7SQ1	5CCK, 7SQ1	1WIO, 7SQ1	1HZH, 5CCK, 7SQ1
Map sharpening *B* factor (Å^2)	147.4	69.3	81.2	90.7	64.3	60.8	106.8	91.4
**Model composition**								
Non-hydrogen atoms	22,481	28,596	26,442	26,337	18,829	16,454	14,673	288,21
Protein residues	2,508	3,354	3,102	3,117	2,240	1,961	1,791	3,522
Ligands	202	173	156	135	89	71	33	100
**R.m.s. deviations**								
Bond lengths (Å)	0.011	0.012	0.012	0.011	0.012	0.011	0.011	0.011
Bond angles (°)	1.230	1.148	1.132	1.155	1.191	1.147	1.033	1.109
**Validation**								
MolProbity score	0.89	0.66	0.64	0.65	0.73	0.76	0.67	0.65
Clashscore	1.47	0.45	0.40	0.41	0.70	0.86	0.48	0.42
Poor rotamers (%)	0.00	0.07	0.07	0.00	0.15	0.17	0.00	0.00
EMRinger score	1.57	3.26	3.25	3.72	2.53	1.62	3.79	1.89
**Ramachandran plot**								
Favored (%)	99.31	98.88	98.82	99.22	99.18	99.22	99.48	99.19
Allowed (%)	0.69	1.12	1.12	0.78	0.82	0.78	0.52	0.81
Disallowed (%)	0.00	0.00	0.00	0.00	0.00	0.00	0.00	0.00

Supplementary Table 2 | Cryo-EM data collection, refinement and validation statistics.
