# Supplementary material for: Conformational landscape of HIV-1 Env from closed to fully open
Source: Nat Commun. 2026 Feb 24;17:4273. doi: 10.1038/s41467-026-69921-z (PMC13168245; doi:10.1038/s41467-026-69921-z)
Supplement: Supplementary file 2 — Description of Additional Supplementary Files [file 41467_2026_69921_MOESM2_ESM.pdf]

Description of Additional Supplementary Files

Supplementary Movie 1 | Direct molecular interaction between b12 and 3BC315. The heavy chain framework region of b12 interacts with the constant light chain domain of 3BC315. 3D variability analysis of our AMC008-b12-3BC315 cryo-EM data reveals a stable interaction throughout the data whereas b12 and 3BC315 interact transiently but in a similar fashion at the two other protomer positions. b12 heavy chain in dodger blue, b12 light chain in light sky blue, 3BC315 heavy chain in forest green, 3BC315 light chain in yellow green, gp41 in orange and gp120 in grey.

Supplementary Movie 2 | Direct Conformational Change from Closed to Occluded Open Env leads to V2 clashes. Linear conformational interpolation from our closed state AAA Env (AMC008-VRC01-35O22) to our open state CDD Env (AMC008-b12-3BC315) reveals massive steric clashes between amino acid positions 163-168 (chartreuse) of the V2 region (otherwise red). gp120 is shown in light gray and gp41 in slate gray.

Supplementary Movie 3 | Conformational Change from Closed over Moderately Open to Occluded Open Env. Conformational interpolation from our closed state AAA Env (AMC008-VRC01-35O22) over our moderately open state BBB Env (AMC008-b12) to our open state CDD Env (AMC008-b12-3BC315) relieves the V2 clashes observed in Movie 1. Thus, moderately open state BBB Env is instrumental to understanding the Env conformational path from closed to open conformations. Coloration as in Movie 1.
